# Supplementary material for: Minimum inhibitory concentrations of rifampin and isoniazid among multidrug and isoniazid resistant Mycobacterium tuberculosis in Ethiopia
Source: PLoS One. 2022 Sep 13;17(9):e0274426. doi: 10.1371/journal.pone.0274426 (PMC9469996; doi:10.1371/journal.pone.0274426)
Supplement: S1 Table — No growth indicates Mtb did not grow; not retrieved indicates the Mtb isolate could not be located and therefore, subculture was not performed; insufficient growth indicates that the colony count was between 3 and 10. (DOCX) [file pone.0274426.s001.docx]

Supplementary Table 1: Subculture growth characteristics of multidrug resistant (MDR), rifampin resistant (RR) and isoniazid (INH) resistant *Mycobacterium tuberculosis* isolates in newly diagnosed and previously treated TB patients

| Subculture growth characteristics | MDR(N=67) | | RR(N=5) | | INH resistant (N=70) | |
| --- | --- | --- | --- | --- | --- | --- |
|  | Newly diagnosed | Previously treated | Newly diagnosed | Previously treated | Newly diagnosed | Previously treated |
| Pure culture and MIC tested | 20 | 26 | 1 | 1 | 19 | 7 |
| Culture positive with contamination | 2 | 2 | 1 | 0 | 0 | 0 |
| No growth on subculture | 1 | 2 | 1 | 0 | 6 | 4 |
| Not retrieved | 3 | 2 | 1 | 0 | 4 | 4 |
| Culture positive/ insufficient growth and MIC not done | 3 | 6 | 0 | 0 | 22 | 4 |

MIC: minimum inhibitory concentration; No growth indicates *Mtb* did not grow; not retrieved indicates the *Mtb* isolate could not be located and therefore, subculture was not performed; insufficient growth indicates that the colony count was between 3 and 10.
