# Supplementary material for: Minimum inhibitory concentrations of rifampin and isoniazid among multidrug and isoniazid resistant Mycobacterium tuberculosis in Ethiopia
Source: PLoS One. 2022 Sep 13;17(9):e0274426. doi: 10.1371/journal.pone.0274426 (PMC9469996; doi:10.1371/journal.pone.0274426)
Supplement: Attachment — Submitted filename: Minimum inhibitory concentrations of rifampin and isoniazid among multidrug and isoniazid resistant Mycobacterium tuberculosis isolates.docx [file pone.0274426.s002.docx]

# Title: Minimum inhibitory concentrations of rifampin and isoniazid among multidrug and isoniazid resistant *Mycobacterium tuberculosis* isolates

Muluwork Getahun^1,2^, Henry M Blumberg^3^, Gobena Ameni^4,5^, Dereje Beyene^2^, and Russell R Kempker^3^

1:- Ethiopian Public Health Institute

2:- Addis Ababa University, Department of Microbial, Cellular and Molecular Biology

3:- Emory University School of Medicine, Department of Medicine, Division of Infectious Diseases, Atlanta, Georgia, USA

4:- Aklilu Lemma Institute of Pathobiology, Addis Ababa University, PO Box 1176, Addis Ababa, Ethiopia

5. Department of Veterinary Medicine, College of Agriculture and Veterinary Medicine, United Arab Emirates University, PO Box 15551, Al Ain, United Arab Emirates University

Corresponding author: Muluwork Getahun

Ethiopian Public Health Institute

P.O. Box 1242

Addis Ababa, Ethiopia

Mail: [mimishaget@yahoo.com](mailto:mimishaget@yahoo.com)

# Abstract

**Background:** Traditionally, single critical concentrations of drugs are utilized for *Mycobacterium tuberculosis* (*Mtb*) drug susceptibility testing (DST); however, the level of drug resistance can impact treatment choices and outcomes. We assessed the minimum inhibitory concentrations (MICs) of multidrug or rifampin resistant (MDR/RR) TB and isoniazid (INH) resistant *Mtb* isolates to determine the quantification of drug resistance among key anti-tuberculosis drugs.

**Method:** The study was conducted on stored *Mtb* isolates collected as part of a national drug resistance survey in Ethiopia. MIC values were determined using Sensititre^TM^ MYCOTB plates. A line probe assay (MTBDR*plus*) was also performed to identify genetic determinants of resistance for all isolates.

**Result:** MIC testing was performed on 74 *Mtb* isolates including 46 MDR, 2 RR and 26 INH phenotypically resistant isolates as determined by the Löwenstein Jensen (LJ) method. Four (15%) INH resistant *Mtb* isolates were detected as borderline rifampin resistance (MIC=1 µg/ml) using MYCOTB MIC plates and no rifampin resistance mutations were detected by LPA. Among the 48 MDR/RR TB cases, 9 (19%) were rifabutin susceptible (MIC was between ≤0.25 and 0.5µg/ml). Additionally, the MIC for isoniazid was between 2-4 µg/ml (moderate resistance) for 58% of MDR TB isolates and 95.6% (n=25) of the isolates had mutations at the *katG* gene.

**Conclusion:** Our findings suggest a role for rifabutin treatment in a subset of RR TB patients, thus potentially preserving an important drug class. The high proportion of moderate level INH resistant among MDR *Mtb* isolates indicates the potential benefit of high dose isoniazid treatment in a high proportion of *katG* gene harboring MDR *Mtb* isolates.

Key words: minimum inhibitory concentration, MDR/RR, isoniazid, rifabutin

**Background**

The effective management of drug resistant tuberculosis (TB) relies on the accurate detection of resistance to guide appropriate treatment (1). Drug susceptibility testing (DST) is an important step in the design of effective treatment regimens as it guides clinical management of individual patients and is essential for TB control program planning (2). Traditionally, a single critical concentration (CC) per drug has been utilized to determine if *Mycobacterium tuberculosis* (*Mtb*) isolates are resistant or susceptible to an individual drug; however, this approach limits the ability to detect borderline resistance and can result in resistance misclassification. Minimum inhibitory concentration (MIC) testing provides quantitative results that may help guide the therapeutic decision-making process (3, 4). High level drug resistance indicates a drug is unlikely to have any clinical benefit; on the other hand, low-level drug resistance indicates a possible benefit of a drug either with standard or increased dosing or from a different drug with the same class (5, 6).

The Sensititre^TM^ MYCOTB plate (Trek Diagnostic Systems, Thermo Fisher Scientific, USA) was developed to determine the MICs of key first- and second-line TB drugs (3, 7, 8). Previous reports have found the Sensititre^TM^ MYCOTB to be accurate method for performing DST for both first- and second-line anti-TB drugs and result would be available within 14 days (3, 7, 8). The results allow the clinician to adjust dose or choose among the best available alternatives to optimize therapy (6, 8, 9). To our knowledge, in Ethiopia there is no previous data on MIC of key first line anti-TB drugs for *Mtb* isolates including multidrug or rifampin resistant (MDR/RR) and isoniazid (INH) resistant isolates. To help fill this knowledge gap, we conducted a study to determine MIC values of MDR/RR TB and INH resistance *Mtb* isolates collected from population-based drug resistance survey. The overall goal of our study is to provide information about the proportion of TB cases with low, moderate, and high-level resistance to INH and the proportion of rifabutin susceptible among RR *Mtb* isolates that could potentially benefit in MIC tailored regimen choice.

**Methods**

We utilized stored *Mtb* isolates to profile the MIC values of MDR/RR TB and INH resistant *Mtb* isolates. The study population has been described in detail previously(10). Briefly, we utilized stored MDR/RR and INH resistant *Mtb* isolates collected for a national drug resistance survey (DRS) conducted between November 2011 and June 2013. The DRS enrolled all TB suspects with sputum smear positive samples from 32 health facilities throughout Ethiopia. The 32 health facilities were purposely selected to include facilities in each region throughout the country and to provide a representative sample of Ethiopia. Phenotypic DST was performed on all *Mtb* isolates using the indirect proportion Jensen (LJ) method for first-line drugs including INH (0.2 μg/ml) and rifampin (RIF) (40 μg/ml). Interpretation of results was based on the proportion of the growth on control and drug containing media (11, 12). The DRS found 67 MDR, 5 RR and 70 INH resistant *Mtb* isolates. Among these isolates, we selected 74 *Mtb* isolates (46 MDR, 2 RR and 26 INH resistant TB) based on subculture growth characteristics and availability of MIC plates (Table 1). The study obtained ethical approval from the Addis Ababa University Ethics Committee.

***Line Probe Assay***

# All stored MDR/RR and INH isolates were under went with LPA testing. The Genotype MTBDR*plus* (Hain Lifescience, Nehren, Germany, version 2) assay was performed to evaluate for genetic determinants of resistance to rifampin and isoniazid. Extraction, amplification, detection, and result interpretation were performed as per the manufacturer’s instructions (13).

# *Minimum Inhibitory Concentration*

After thawing frozen *Mtb* isolates, we sub cultured on Middlebrook 7H11 agar medium. Sensititre^TM^ MYCOTB MIC plates were utilized for MIC testing for all included *Mtb* isolates. The MYCOTB plate contains lyophilized antibiotics of three key first-line drugs including isoniazid (0.25-4 µg/ml), rifampin (0.25-8 µg/ml) and rifabutin (0.25-8 µg/ml). MIC testing was performed according to the manufacturer’s recommendation. Briefly, colonies were scraped, and a 0.5 McFarland standard was prepared using a saline-Tween solution. After 15 minutes, 100 microliters were transferred to 11ml of Middlebrook 7H9 broth containing oleic acid albumin-dextrose-catalase and vortexed for 30s. Each well of the MIC plate was inoculated with 100 microliters of the reconstituted sample aliquot. Plates were then covered with plastic seals and incubated at 37°C, and visually monitored for growth at days 1, 2, 7, 10, 14 and 21. The MIC result was recorded on days 10 and subsequently on 21 if growth was not sufficient for interpretation. Blood agar and Middlebrook 7H10 plates were also inoculated with suspension to check contamination and read after 24 and 48 hours.

We classified *Mtb* isolates as either susceptible or resistant based on reported CCs (3, 4, 7, 9, 14). The lowest concentration with no visible growth was viewed using a magnified mirror and recorded as the MIC value. *Mtb* isolates were defined as resistant if the MIC was greater than the CC or susceptible if the MIC was less than or equal to the CC. For INH, we categorized into the following 3 groups based on MIC values: low level resistance (≤0.25µg/ml to 0.5µg/ml), moderate resistance (1µg/ml≥ MIC ≤4µg/ml) and high level resistance (MIC>4µg/ml). The upper value of moderate level resistance were defined based on a clinical trial report in which patients with such levels of resistance were successfully treated with high dose INH(15).

***Statistical analysis***

Descriptive statistics were used to describe the MIC values of MDR, RR and INH resistant *Mtb* isolates.

**Results**

***Study isolates and patients***

A total of 74 *Mtb* isolates from unique patients including 46 MDR, 2 RR and 26 INH phenotypically resistant isolates had MIC testing using MYCOTB Sensititre^TM^ plates performed. Among the 74 unique individuals from which the samples came from, 58% were male and the mean age was 30 years. Forty six percent of patients (n=34) had a history of prior anti-TB treatment with first-line drugs. Ninety five percent (n=70) of the patients had human immunodeficiency virus (HIV) testing performed and 33% (n=23) were HIV positive.

# *MIC Testing*

Among the 46 MDR, 2 RR and 26 INH resistant *Mtb* isolates, MYCOTB Sensititre^TM^ MIC plate confirmed resistance in 46, 2 and 24 cases respectively (Table 2). Two INH resistance *Mtb* isolates as defined by LJ method were detected as susceptible using Sensititre^TM^ MYCOTB MIC plate (MIC≤0.25 µg/ml) and mutations confirming INH resistance were detected using LPA. Four (15%) INH resistant *Mtb* isolates as defined by LJ method were borderline rifampin resistant TB at CC= 0.5 µg/ml using MYCOTB MIC plates and no rifampin resistance mutations were detected by LPA.

***Rifabutin susceptible RR Mtb***

Among 48 MDR/RR *Mtb* isolates, 9 isolates (19%) were found to be rifabutin-susceptible (MIC≤0.5µg/ml), including 6 from patients with a prior history of TB treatment. The frequency of rifabutin susceptible RR *Mtb* with the corresponding MTBDR*plus* codon were displayed in Table 3. Thirty three percent of the rifampin-resistant and rifabutin-susceptible *Mtb* isolates had mutations detected by the MDRTB*plus* probe in the 513-519 codon regions (Table 3).

***Low and moderate level isoniazid Mtb***

Among 46 MDR *Mtb* isolates, 58% (n=27) had moderate level (MIC=2-4 µg/ml) isoniazid resistance. Most (n=25, 95.6%) moderate level isoniazid resistant *Mtb* isolates had mutations in the *katG* gene; of the remaining two isolates, one had mutations at the *inhA* gene mutations and the other isolates had no mutations detected by the LPA (Table 4). Among 26 INH resistant *Mtb* isolates, 3 (12%) had low level resistance (MIC ≤0.5µg/ml) including 2 with mutations detected by the MTBDR*plus* in the *inhA* gene.

**Discussion**

Utilizing a national representative sample, we carried out the first study evaluating MIC testing of *Mtb* isolates in Ethiopia with results highlighting potential roles for rifabutin and high dose INH treatment in many patients with MDR TB. Our study reports borderline rifampin resistance among INH resistant *Mtb*, low level rifabutin resistance among RR *Mtb* and moderate level isoniazid resistance among *katG* gene harboring MDR *Mtb* isolates. Data on rifabutin susceptible RR *Mtb* would assist in guiding the treatment choice for the RFB-based therapy (16). Additionally, detections of borderline rifampin resistance using Sensititre^TM^ MYCOTB MIC plate which mostly missed using phenotypic methods are vital to interrupt transmission (17-19). Besides, information on the level of INH resistance is important to guide the treatment choice with high dose INH for MDR *Mtb* with *katG* mutations (15).

Rifabutin is an alternative first line [anti-TB](https://en.wikipedia.org/wiki/Antibiotic) drug and active against rifampin resistance for some proportion of strains (20). The presence or absence of cross resistance of rifampin resistance with rifabutin may assist the clinical decision process(21). In our study, 19% of the *Mtb* isolates that were MDR/RR were susceptible to rifabutin; a finding that is in line with previous studies (20, 22). Studies show that the treatment outcomes and the rate of acquired resistance of rifabutin susceptible MDR TB patients can be improved by rifabutin-based therapy (23, 24). A clinical trial on MDR TB patients who had been treated using individualized regimens reports a significantly higher treatment success rate for RFB based therapy (85.7%) than control group (52.4%) (16). Additionally, mutations at codon 514, 516 and 522 are usually associated with RFB susceptibility even though the mutations alone had not always classified such cases (25-27). In our study, we found that 33% rifampin resistant but rifabutin susceptible was detected at codon 513-519; this is in line with previous studies (28, 29). Overall, our finding showed that the MIC of rifabutin susceptible MDR/RR *Mtb* isolates were between ≤0.25 and 0.5µg/ml which indicates the value of MIC determination in detecting those cases than the traditional DST which uses a single CC as well as frontline genotypic methods(30).

Borderline rifampin resistance is mostly associated with low-level rifampin resistance and can be missed by growth-based methods (17-19). Our finding showed that 15% INH resistant *Mtb* isolates as defined by LJ method were borderline rifampin resistance at CC= 0.5 µg/ml (MIC=1 µg/ml) using Sensititre^TM^ MYCOTB MIC plate. We did not detect rifampin mutations using LPA suggesting the mutation might occur outside of rifampicin resistance-determining region. The WHO review indicates that a couple of rifampin borderline resistance was detected as susceptible using LJ method at the current CC (40 mg/L). Compare to LJ method, 36% of mutations at codon I572F and 28 % of mutation at the six rifampicin resistance-determining region were detected as susceptible using LJ method (30). The treatment outcome of patients with borderline rifampin resistance has been associated with treatment failures (31) and WHO recommends a MDR-TB regimen for their treatment (30). Our findings indicate the importance of MIC in detecting borderline rifampin resistance which is mostly difficult to detect using phenotypic and frontline genotypic methods (17-19, 30).

World Health Organization recommended the inclusion of high dose isoniazid in MDR TB regimen to increase the treatment success(32). Although the clinical efficacy of high dose isoniazid is not fully understood (33, 34). In our study, the MIC of isoniazid was moderate level (1µg/ml≥ MIC ≤4µg/ml) for just over half of all MDR TB isolates, which is consistent with other studies (35, 36). We found that 95.6% of moderate level isoniazid resistant TB had mutations in the *katG* gene and only one isolate had mutations at the *inhA* gene. A previous study reports MDR TB patients with *inhA* mutation have MIC value ranges from 0.05-4µg/ml and that they had comparable early bactericidal activity as sensitive strains. The high dose treatment(10–15 mg/kg daily) procedure a similar early bactericidal activity for participant with *inhA* resistance TB compared to isoniazid sensitive TB participant who were treated with standard dose (5mg/kg daily) (15). As noted, the treatment outcome of low to moderate level isoniazid resistance with high dose isoniazid treatment would be increased if aligned with MIC results (37-39). Our finding suggests a high proportion of *katG* harboring MDR TB patients could benefit from high dose INH if MIC testing is performed.

Limitations of our study include the following: First, the MIC was performed in selected isolates especially for INH resistance. Additionally, treatment data were not available precluding us from evaluating outcomes based on certain phenotypic and genetic resistance.

# Conclusion

We profile the MIC of rifampin, rifabutin and isoniazid using MYCOTB Sensititre^TM^ MIC plate. Our findings suggest a potential role for rifabutin treatment in certain patients with RR TB and further studies are needed to explore the utility of providing a rifabutin for RR TB patients. Moreover, over half of MDR TB isolates had moderate level isoniazid resistant indicates the potential benefit of high dose isoniazid treatment in a high proportion of *katG* gene harboring MDR *Mtb* isolates if aligned with MIC values. Furthermore, additional data on MIC testing is important to guiding management and allowing for personalized treatment in low-income countries like Ethiopia.

**Conflicts of interest**

We declare there is no conflict of interest.

**Funding**

This work was supported in part by the Ethiopian Public Health Institute and the National Institutes of Health Fogarty International Center (D43 TW009127).

**Authors' contributions**

MG conceived, developed the protocol, conducted the study, and drafted the manuscript. HMB and RRK advised the protocol development and reviewed and edited the draft manuscript. DB and GA reviewed and edited the draft manuscript. HM and GD conducted laboratory analysis and quality control. All authors read the manuscript.

**References**

1.	World Health Organization. Technical manual for drug susceptibility testing of medicines used in the treatment of tuberculosis. 2018.

2.	World Health Organization. Implementing tuberculosis diagnostics: policy framework. 2015.

3.	Hall L, Jude KP, Clark SL, Wengenack NL. Antimicrobial susceptibility testing of Mycobacterium tuberculosis complex for first and second line drugs by broth dilution in a microtiter plate format. JoVE (Journal of Visualized Experiments). 2011 Jun 24(52):e3094.

4.	Abuali MM, Katariwala R, LaBombardi VJ. A comparison of the Sensititre® MYCOTB panel and the agar proportion method for the susceptibility testing of Mycobacterium tuberculosis. European journal of clinical microbiology & infectious diseases. 2012 May;31(5):835-9.

5.	Böttger EC. The ins and outs of Mycobacterium tuberculosis drug susceptibility testing. Clinical microbiology and infection. 2011 Aug 1;17(8):1128-34.

6.	Mpagama SG, Houpt ER, Stroup S, Kumburu H, Gratz J, Kibiki GS, et al. Application of quantitative second-line drug susceptibility testing at a multidrug-resistant tuberculosis hospital in Tanzania. BMC infectious diseases. 2013 Dec;13(1):1-8.

7.	Lee J, Armstrong DT, Ssengooba W, Park JA, Yu Y, Mumbowa F, et al. Sensititre MYCOTB MIC plate for testing Mycobacterium tuberculosis susceptibility to first-and second-line drugs. Antimicrobial agents and chemotherapy. 2014 Jan;58(1):11-8.

8.	Heysell SK, Ahmed S, Ferdous SS, Khan MS, Rahman SM, Gratz J, et al. Quantitative drug-susceptibility in patients treated for multidrug-resistant tuberculosis in Bangladesh: implications for regimen choice. PLoS One. 2015 Feb 24;10(2):e0116795.

9.	Heysell SK, Pholwat S, Mpagama SG, Pazia SJ, Kumburu H, Ndusilo N, et al. Sensititre MycoTB plate compared to Bactec MGIT 960 for first-and second-line antituberculosis drug susceptibility testing in Tanzania: a call to operationalize MICs. Antimicrobial agents and chemotherapy. 2015 Nov 1;59(11):7104-8.

10.	Getahun M, Ameni G, Mollalign H, Diriba G, Beyene D. Genotypic and Phenotypic Drug Resistant Detection and Prevalence of Heteroresistance in Patients with Isoniazid and Multidrug Resistance Tuberculosis in Ethiopia. IJID Regions. 2022 Jan 2.

11.	Canetti G, Fox W, Khomenko A, et al. Advances in techniques of testing mycobacterial drug sensitivity, and the use of sensitivity tests in tuberculosis control programmes. Bull World Health Organ. 1969;41(1):21-43. Kent PT. Public health mycobacteriology: a guide for the level III laboratory. US department of health and human services, public health service, Centers for Disease Control; 1985.

12.	Kent PT. Public health mycobacteriology: a guide for the level III laboratory. US department of health and human services, public health service, Centers for Disease Control; 1985. World Health Organization. The use of molecular line probe assay for the detection of resistance to isoniazid and rifampicin: policy update. 2016.

13.	World Health Organization. The use of molecular line probe assay for the detection of resistance to isoniazid and rifampicin: policy update. 2016.

14.	Abdel-Rahman SM, Abdel-Latif W, Kholeif H, Erfan D. Evaluation of Sensititre® MycoTB panel for the susceptibility testing of Mycobacterium tuberculosis to first and second lines anti-tuberculosis drugs. J Clin Med Diagn. 2016;6(1):13-9.

15.	Dooley KE, Miyahara S, von Groote-Bidlingmaier F, Sun X, Hafner R, Rosenkranz SL, et al. Early bactericidal activity of different isoniazid doses for drug-resistant tuberculosis (INHindsight): a randomized, open-label clinical trial. American journal of respiratory and critical care medicine. 2020 Jun 1;201(11):1416-24.

16.	Jo KW, Ji W, Hong Y, Lee SD, Kim WS, Kim DS, et al. The efficacy of rifabutin for rifabutin-susceptible, multidrug-resistant tuberculosis. Respiratory medicine. 2013 Feb 1;107(2):292-7.

17.	Miotto P, Cabibbe AM, Borroni E, Degano M, Cirillo DM. Role of disputed mutations in the rpoB gene in interpretation of automated liquid MGIT culture results for rifampin susceptibility testing of Mycobacterium tuberculosis. Journal of clinical microbiology. 2018 May 1;56(5):e01599-17.

18.	Rigouts L, Gumusboga M, de Rijk WB, Nduwamahoro E, Uwizeye C, de Jong B, et al. Rifampin resistance missed in automated liquid culture system for Mycobacterium tuberculosis isolates with specific rpoB mutations. Journal of clinical microbiology. 2013 Aug;51(8):2641-5.

19.	Torrea G, Ng KC, Van Deun A, André E, Kaisergruber J, Ssengooba W, et al. Variable ability of rapid tests to detect Mycobacterium tuberculosis rpoB mutations conferring phenotypically occult rifampicin resistance. Scientific reports. 2019 Aug 14;9(1):1-9.

20.	Farhat MR, Sixsmith J, Calderon R, Hicks ND, Fortune SM, Murray M. Rifampicin and rifabutin resistance in 1003 Mycobacterium tuberculosis clinical isolates. Journal of Antimicrobial Chemotherapy. 2019 Jun 1;74(6):1477-83.

21.	Somoskovi A, Salfinger M. How can the tuberculosis laboratory aid in the patient-centered diagnosis and management of tuberculosis?. Clinics in chest medicine. 2019 Dec 1;40(4):741-53.

22.	Tan Y, Hu Z, Zhao Y, Cai X, Luo C, Zou C, et al. The beginning of the rpoB gene in addition to the rifampin resistance determination region might be needed for identifying rifampin/rifabutin cross-resistance in multidrug-resistant Mycobacterium tuberculosis isolates from Southern China. Journal of clinical microbiology. 2012 Jan;50(1):81-5.

23.	Lee H, Ahn S, Hwang NY, Jeon K, Kwon OJ, Huh HJ, et al. Treatment outcomes of rifabutin-containing regimens for rifabutin-sensitive multidrug-resistant pulmonary tuberculosis. International Journal of Infectious Diseases. 2017 Dec 1;65:135-41.

24.	Li J, Munsiff SS, Driver CR, Sackoff J. Relapse and acquired rifampin resistance in HIV-infected patients with tuberculosis treated with rifampin-or rifabutin-based regimens in New York City, 1997–2000. Clinical infectious diseases. 2005 Jul 1;41(1):83-91.

25.	Zhang Y, Yew WW. Mechanisms of drug resistance in Mycobacterium tuberculosis [State of the art series. Drug-resistant tuberculosis. Edited by CY. Chiang. Number 1 in the series]. The International Journal of Tuberculosis and Lung Disease. 2009 Nov 1;13(11):1320-30.

26.	Somoskovi A, Salfinger M. Commentary: The Race Is On To Shorten the Turnaround Time for Diagnosis of Multidrug-Resistant Tuberculosis. Journal of clinical microbiology. 2015 Dec;53(12):3715-8.

27.	Whitfield MG, Warren RM, Mathys V, Scott L, De Vos E, Stevens W, et al. The potential use of rifabutin for treatment of patients diagnosed with rifampicin-resistant tuberculosis. 2018;73(10):2667-74.

28.	Jagielski T, Bakuła Z, Brzostek A, Minias A, Stachowiak R, Kalita J, et al. Characterization of mutations conferring resistance to rifampin in Mycobacterium tuberculosis clinical strains. Antimicrobial agents and chemotherapy. 2018 Sep 24;62(10):e01093-18.

29.	Jamieson FB, Guthrie JL, Neemuchwala A, Lastovetska O, Melano RG, Mehaffy C. Profiling of rpoB mutations and MICs for rifampin and rifabutin in Mycobacterium tuberculosis. Journal of clinical microbiology. 2014 Jun;52(6):2157-62.

30.	World Health Organization. Technical report on critical concentrations for drug susceptibility testing of isoniazid and the rifamycins (rifampicin, rifabutin and rifapentine).2021.

31.	Williamson DA, Roberts SA, Bower JE, Vaughan R, Newton S, Lowe O, et al. Clinical failures associated with rpoB mutations in phenotypically occult multidrug-resistant Mycobacterium tuberculosis. The international journal of tuberculosis and lung disease. 2012 Feb 1;16(2):216-20.

32.	Falzon D, Schünemann HJ, Harausz E, González-Angulo L, Lienhardt C, Jaramillo E, et al. World Health Organization treatment guidelines for drug-resistant tuberculosis, 2016 update. 2017;49(3).

33.	World Health Organization. WHO consolidated guidelines on drug-resistant tuberculosis treatment. World Health Organization; 2019.

34.	World Health Organization. WHO treatment guidelines for isoniazid-resistant tuberculosis: supplement to the WHO treatment guidelines for drug-resistant tuberculosis. World Health Organization; 2018. Report No.: 9241550074.

35.	Schönfeld N, Bergmann T, Vesenbeckh S, Mauch H, Bettermann G, Bauer TT, et al. Minimal inhibitory concentrations of first-line drugs of multidrug-resistant tuberculosis isolates. Lung India: Official organ of Indian chest society. 2012 Oct;29(4):309.

36.	Schaaf HS, Victor TC, Engelke E, Brittle W, Marais BJ, Hesseling AC, et al. Minimal inhibitory concentration of isoniazid in isoniazid-resistant Mycobacterium tuberculosis isolates from children. European Journal of Clinical Microbiology & Infectious Diseases. 2007 Mar;26(3):203-5.

37.	Moulding TS. Should isoniazid be used in retreatment of tuberculosis despite acquired isoniazid resistance?. American Review of Respiratory Disease. 1981 Mar;123(3):262-4.

38.	Lempens P, Meehan CJ, Vandelannoote K, Fissette K, de Rijk P, Van Deun A, et al. Isoniazid resistance levels of Mycobacterium tuberculosis can largely be predicted by high-confidence resistance-conferring mutations. Scientific reports. 2018 Feb 19;8(1):1-9.

39.	Lange C, Abubakar I, Alffenaar JW, Bothamley G, Caminero JA, et al. Management of patients with multidrug-resistant/extensively drug-resistant tuberculosis in Europe: a TBNET consensus statement.

Table 1: Subculture growth characteristics of multidrug resistant (MDR), rifampin resistant (RR) and isoniazid (INH) resistant *Mycobacterium tuberculosis* isolates in newly diagnosed and previously treated TB patients

| Subculture growth characteristics | MDR(N=67) | | RR(N=5) | | INH resistant (N=70) | |
| --- | --- | --- | --- | --- | --- | --- |
|  | Newly diagnosed | Previously treated | Newly diagnosed | Previously treated | Newly diagnosed | Previously treated |
| Pure culture and MIC tested | 20 | 26 | 1 | 1 | 19 | 7 |
| Culture positive with contamination | 2 | 2 | 1 | 0 | 0 | 0 |
| No growth on subculture | 1 | 2 | 1 | 0 | 6 | 4 |
| Not retrieved | 3 | 2 | 1 | 0 | 4 | 4 |
| Culture positive/ insufficient growth and MIC not done | 3 | 6 | 0 | 0 | 22 | 4 |

MIC: minimum inhibitory concentration

Table 2: Minimum inhibitory concentration of rifampin, rifabutin and isoniazid among multidrug resistant (MDR), rifampin resistant (RR) and isoniazid (INH) resistant *Mycobacterium tuberculosis* isolates

| Drugs | DST result | MIC in µg/ml | Total N | Total % | Newly diagnosed, N | Retreatment, N |
| --- | --- | --- | --- | --- | --- | --- |
| Rifampin | MDR | >8 | 46 | 100 | 21 | 27 |
|  | RR TB | >8 | 2 | 100 | 1 | 1 |
|  | INH resistant | 0.5 | 4 | 15 | 4 | 0 |
|  |  | ≤0.25 | 22 | 85 | 15 | 7 |
| Rifabutin | MDR | >8 | 18 | 39 | 4 | 14 |
|  |  | 8 | 7 | 15 | 3 | 4 |
|  |  | 4 | 9 | 20 | 9 | 0 |
|  |  | 2 | 2 | 4 | 1 | 1 |
|  |  | 1 | 2 | 4 | 1 | 1 |
|  |  | 0.5 | 1 | 2 | 0 | 1 |
|  |  | ≤0.25 | 7 | 15 | 2 | 5 |
|  | RR TB | 2 | 1 | 50 | 0 | 1 |
|  |  | ≤0.25 | 1 | 50 | 1 | 0 |
|  | INH resistance | ≤0.25 | 26 | 100 | 19 | 7 |
| Isoniazid | MDR | >4 | 19 | 41 | 8 | 11 |
|  |  | 4 | 25 | 54 | 10 | 15 |
|  |  | 2 | 2 | 4 | 2 | 0 |
|  | INH resistance | >4 | 9 | 35 | 1 | 8 |
|  |  | 4 | 9 | 35 | 5 | 4 |
|  |  | 2 | 5 | 8 | 4 | 1 |
|  |  | 0.5 | 1 | 4 | 1 | 0 |
|  |  | ≤0.25 | 2 | 8 | 1 | 1 |

DST: drug susceptibility testing

Table 3: MTBDR*plus* probe and minimum inhibitory concentration results of rifabutin susceptible and rifampin resistant *Mycobacterium tuberculosis* isolates (n=9)

| Drug susceptibility test result | MIC in µg/ml | Number of cases | Target region and MTBDR*plus* probe (Codon) | History of treatment | |
| --- | --- | --- | --- | --- | --- |
|  |  |  |  | New | Retreatment |
| MDR | ≤0.25 | 2 | W3W4 (513-519) | 1 | 1 |
|  |  | 4 | W7 (525-529) | 0 | 4 |
|  |  | 1 | W7MUT2A(525-529) | 1 | 0 |
|  | 0.5 | 1 | W3W4MUT1(513-519) | 0 | 1 |
| RR | ≤0.25 | 1 | W7(525-529) | 1 | 0 |

Table 4: Frequency of isoniazid resistance *Mycobacterium tuberculosis* isolates with minimum inhibitory concentration and MTBDR*plus* probe result

| DST result | MIC in µg/ml | Total | Target region and MTBDR*plus* probe | | | | |
| --- | --- | --- | --- | --- | --- | --- | --- |
|  |  |  | Susceptible | inhAW1MUT1 | katGW1 | katGW1MUT1 | katGMUT1 |
| INH resistant | ≤0.25 | 2 | 0 | 1 | 0 | 0 | 1 |
|  | 0.5 | 1 | 0 | 1 | 0 | 0 | 0 |
|  | 2 | 5 | 2 | 0 | 0 | 3 | 0 |
|  | 4 | 9 | 0 | 0 | 0 | 9 | 0 |
|  | >4 | 9 | 0 | 0 | 0 | 8 | 1 |
| MDR | 2 | 2 | 0 | 0 | 0 | 2 | 0 |
|  | 4 | 25 | 1 | 1 | 2 | 21 | 0 |
|  | >4 | 19 | 1 | 0 | 0 | 17 | 1 |

DST: drug susceptibility testing
