# Supplementary material for: Minimum inhibitory concentrations of rifampin and isoniazid among multidrug and isoniazid resistant Mycobacterium tuberculosis in Ethiopia
Source: PLoS One. 2022 Sep 13;17(9):e0274426. doi: 10.1371/journal.pone.0274426 (PMC9469996; doi:10.1371/journal.pone.0274426)
Supplement: Attachment — Submitted filename: Review_ethiopia.docx [file pone.0274426.s003.docx]

The manuscript entitled “Minimum inhibitory concentrations of rifampicin and isoniazid among multidrug and isoniazid resistant Mycobacterium tuberculosis isolates” has been reviewed and I would like to comment on the overall hypothesis and its execution. Authors here had tried to demonstrate that how important is the dose spectrum of the current anti-TB drugs. And how this can affect the treatment choices and outcome. This is a very serious concern where it is difficult to deicide on clinical breakpoints of these drugs for effective treatment outcome. My comments are below which would help in better understanding of the current study and if fulfilled, I would recommend acceptance.

1) My major concern is the use of LPA which; by now have several modifications after 2011 for determining the resistance of drugs. However, authors use a latest version of LPA which was modified in 2017/2018 but I am not sure if there are additional guidelines by Hains in 2019 to calculate the resistance of these drugs? Author should clarify this and give a detail about it.
2) What is the concentration used for sub- culturing these strains on 7H11 plates?
3) What is the difference between no growth on subculture and not retrieved group?
4) I am curious to know if there are inoculum differences when the strains were scrapped off from the 7H11 plate to resuspend for McFarland standard? For example, how much cfu/ml would it be in the borderline strains?
5) The detection rate of different type of mutations, for example mutations at different SNP’s have authors considered for detection rate, when determining the type of mutation?
6) I understand the authors mentioned about the data regarding treatment is not accessible but important to know that the mutation outside Rifampicin determining region in those two borderline mutant strains is a result of treatment failure/lost follow up.
7) Is there any possibility of predicting that mutations will occur in which region and whether they are correlated with the MIC? Are there any models? Authors may give description about it.
8) Are the suspects in the newly diagnosed category have a known TST and IGRA status?
9) I am always skeptical about using high concentrations of drugs, authors mention the use high concentrations of INH for treatment. Unfortunately, mechanistically it remains poorly understood. Also, it is important to note that INH is poorly penetrant and its possible to have side effects and emergence of persisters later. However, I do not deny that there are studies done but those TB treated subjects I am not sure if they followed up which remains a cause of concern.
