# Supplementary material for: Minimum inhibitory concentrations of rifampin and isoniazid among multidrug and isoniazid resistant Mycobacterium tuberculosis in Ethiopia
Source: PLoS One. 2022 Sep 13;17(9):e0274426. doi: 10.1371/journal.pone.0274426 (PMC9469996; doi:10.1371/journal.pone.0274426)
Supplement: Attachment — Submitted filename: Response to Reviewers.docx [file pone.0274426.s004.docx]

Dear PLoS One,

We appreciate the time and effort of the editor and reviewers in providing a detailed review of our manuscript. We have provided a point-by-point response below to outline how we have addressed the editor and reviewers’ comments. We thank you for the consideration of our work,

Sincerely,

Muluwork Getahun on behalf of the study team

**Response to Reviewers**

Reviewer #1:

**1. Comment**: This article on "Minimum inhibitory concentrations of rifampin and isoniazid among multidrug and isoniazid resistant Mycobacterium tuberculosis isolates" was very well written, concise and easy to follow. In the context of Ethiopia, this type of study is highly significant. Since there was significant number of patients were identified with HIV positive, it was not discussed about the possible outcome/impact of HIV on MDR TB. However, please review the attached file for detailed comments.

**Response**: Thank you for this comment. To highlight the topics of HIV and MDR-TB we have made revisions in the revised manuscript as follows.

Page 9 line 187-189: “Among 48 MDR/RR Mtb isolates, 9 isolates (19%) were found to be rifabutin-susceptible (MIC≤0.5µg/ml), including 6 patients with a prior history of TB treatment and 4 from persons living with HIV.”

Page 10 line 214-215: “Rifabutin is considered as an alternative treatment option in place of rifampin in some persons with HIV and TB due to fewer drug-drug interactions with certain antiretroviral drugs.”

Page 10 line 226-227: “Another clinical trial indicates that TB-HIV co-infected patients who takes rifabutin twice a week had low relapse rate (1 of 20) compared to those on rifampin-based therapy (8 of 9) (Burman et al., 2006).”

Reviewer #2:

Major comments:
**2. Comment**: Study sample size: The manuscript provides drug susceptibility data for 74 samples isolated from 32 health facilities from different locations of Ethiopia. However, the given sample size is far too small for making a representation of the TB drug susceptibility profile of Ethiopia. The authors could consider increasing the sample size.

**Response**: We included all the available MDR/RR *Mtb* isolates, all previously treated isoniazid resistant *Mtb* and 40% of newly diagnosed isoniazid resistant *Mtb* isolates that were collected from the national drug resistance survey*.* The remaining 22 newly diagnosed isoniazid Mtb isolates did not undergo testing with MIC due to the shortage of MIC plates

**3. Comment**: Based on their observation, the authors recommend considering the use of rifabutin and high dose INH for the treatment of MDR-TB. It seems plausible. However, despite the available references on the use of rifabutin and high dose INH for treatment of MDR-TB, I feel that the sample size of the current study and the fraction (9 out of 48 MDR isolates, 19%) is yet very small to conclude this point.

**Response**: We agree with the reviewer’s comment. While our study highlights the potential role for rifabutin for MDR TB patients the small sample size precludes broad generalizability. Based on the reviewer’s comment and to make this point clear, we have added in the following sentence to the limitations paragraph in the revised manuscript.

Page 12 line 264-267: “Therefore, a large scale study that includes treatment outcome and adverse events would provide additional needed data to inform evidence-based guidelines on the use of rifabutin and high dose isoniazid in the treatment of MDR-TB. “

**4. Comment:** The title of the manuscript looks very general. I would suggest revising the manuscript title to include the specific geographical location that is the country name.

**Response:** Based on the reviewer’s comment, we have revised the title of the manuscript to include the location of the work. The new title of the manuscript (line 1, page 1) is “Minimum inhibitory concentrations of rifampin and isoniazid among multidrug and isoniazid resistant *Mycobacterium tuberculosis* in Ethiopia”

Minor comments:

**5. Comment**. Add the full form of RFB in the first place

**Response:** Thank you for this comment. In response to this comment, we are now using “rifabutin” rather than RFB throughout the manuscript.

**6. Comment** L94: Underwent

**Response**: We have revised this sentence in the revised manuscript. Page 6 line 130: “All stored MDR/RR and INH-resistant *Mtb* isolates had LPA testing performed.”

**7. Comment L100**: Subcultured

Response: We have revised this sentence in the revised manuscript. Page 6 line 136-137: “After thawing frozen *Mtb* isolates, 100 µl of vortexed allocate was subcultured on to Middlebrook 7H11 agar medium.

Reviewer #3:

**8. Comment**: My major concern is the use of LPA which; by now have several modifications after 2011 for determining the resistance of drugs. However, authors use a latest version of LPA which was modified in 2017/2018 but I am not sure if there are additional guidelines by Hains in 2019 to calculate the resistance of these drugs? Author should clarify this and give a detail about it.

**Response**: Our study utilized the latest version of the Hains LPA test, and there have been no further modifications since that time.

**9. Comment**: What is the concentration used for sub-culturing these strains on 7H11 plates?

**Response:** We used 100 µl of vortexed *Mtb* isolates, and we have highlighted this in the manuscript as outlined below.

Page 6 line 136-137: “After thawing frozen *Mtb* isolates, 100 µl of vortexed allocate was subcultured on to Middlebrook 7H11 agar medium.”

**10. Comment**: What is the difference between no growth on subculture and not retrieved group?

**Response:** No growth indicates that the subculture was performed but *Mtb* did not grow. While, not retrieved indicates the isolates were not located and available and therefore, a subculture was not performed. We have made the following revision in the revised manuscript:

Page 26 line 499-501: “No growth indicates *Mtb* did not grow; not retrieved indicates the *Mtb* isolate could not be located and therefore, subculture was not performed; insufficient growth indicates that the colony count was between 3 and 10.”

**11. Comment**: I am curious to know if there are inoculum differences when the strains were scrapped off from the 7H11 plate to resuspend for McFarland standard? For example, how much cfu/ml would it be in the borderline strains?

**Response:** We used a 0.5 McFarland standard for all isolates which equates to CFU/ml of 1.5 x 10^8^. This ensured that all inoculum sizes were the same.

**12. Comment:** The detection rate of different type of mutations, for example mutations at different SNP’s have authors considered for detection rate, when determining the type of mutation?

**Response:** We have outlined the mutations identified by LPA in Table 2 and have revised the following sentences:

Page 9 line 185-186: The MICs of *Mtb* isolates to rifabutin and the corresponding mutation profiles are summarized in Table 2. The identified mutation profiles are described under comment 14.

**13. Comment:** I understand the authors mentioned about the data regarding treatment is not accessible but important to know that the mutation outside Rifampicin determining region in those two borderline mutant strains is a result of treatment failure/lost follow up.

**Response:** The borderline mutant strains were present among patients with newly diagnosed INH-resistant TB. The following revision was made based on the reviewer’s comment:

Page 8 line 182-183: The *Mtb* isolates with borderline rifampin resistant were recovered from patients with newly diagnosed TB.

**14. Comment:** Is there any possibility of predicting that mutations will occur in which region and whether they are correlated with the MIC? Are there any models? Authors may give description about it.

**Response:** Mutations at codon 530-533 likely had a higher MIC to rifabutin. The information is described in the revised manuscript as follows:

Page 9 line 186-187: Sixty two percent of the mutations occurred at codon 530-533 and 93% of rifabutin resistant *Mtb* isolate had MIC ≥ 4µg/ml.

Page 9 line 190-192: Six (67%) of 9 rifampin-resistant and rifabutin-susceptible *Mtb* isolates were classified as rifampin resistance due to the absence of wild-type probes only.

**15. Comment**: Are the suspects in the newly diagnosed category have a known TST and IGRA status?

**Response:** Thank you for this comment. Unfortunately, we did not have this clinical information on TST and IGRA results. Additionally, it should be noted that the TST and IGRA are not routinely performed among persons with suspected TB in Ethiopia.

**16. Comment:** I am always skeptical about using high concentrations of drugs, authors mention the use high concentrations of INH for treatment. Unfortunately, mechanistically it remains poorly understood. Also, it is important to note that INH is poorly penetrant and its possible to have side effects and emergence of persisters later. However, I do not deny that there are studies done but those TB treated subjects I am not sure if they followed up which remains a cause of concern.

**Response:** We appreciate the reviewer’s comments and agree with this concern. Regardless of MIC testing results, high dose isoniazid is often part of an empiric MDR TB treatment regimen in low income countries such as Ethiopia. As noted, the treatment outcome of low to moderate level isoniazid resistance with high dose isoniazid treatment would be increased if the treatment is guided by MIC results (Moulding, 1981, Lange et al., 2014). Our study highlights the potential role of the use of high dose INH treatment in many patients with MDR TB if an MIC test guided approach is implemented.

Additionally, a large scale phase II clinical trial on “High-Dose Isoniazid Among Adult Patients With Different Genetic Genetic Variants of INH-Resistant Tuberculosis (TB)” would provide the needed evidence. (<https://clinicaltrials.gov/ct2/show/NCT01936831?cond=isoniazid&draw=3&rank=24>)

Based on the reviewer’s comment, the manuscript has been revised as follows:

Page 12 line 264-267: Therefore, a large scale study that includes treatment outcome and adverse events would provide additional needed data to inform evidence-based guidelines on the use of rifabutin and high dose isoniazid in the treatment of MDR-TB.
